# Burden of treatment as a measure of healthcare quality: An innovative approach to addressing global inequities in multimorbidity

**DOI:** 10.1371/journal.pgph.0000484

**Published:** 2022-05-10

**Authors:** Andrea Gaspar, J. Jaime Miranda

**Affiliations:** 1 CRONICAS Center for Excellence in Chronic Diseases, Universidad Peruana Cayetano Heredia, Lima, Peru; 2 School of Medicine, Universidad Peruana Cayetano Heredia, Lima, Peru; 3 The George Institute for Global Health, University of New South Wales, Sydney, Australia; 4 Faculty of Epidemiology and Population Health, London School of Hygiene and Tropical Medicine, London, United Kingdom; Global Malaria Programme, World Health Organization, SWITZERLAND

Global evidence shows that system quality failures are responsible for more deaths than those caused by HIV, tuberculosis, and malaria combined [1]. In low- and middle-income countries (LMICs), the effects of poor quality care are particularly profound. Nearly 60% of the 8 million preventable deaths that occur yearly in LMICs are due to quality deficits [2]. These span many sectors of healthcare, including primary care, where deficiencies in disease prevention, care coordination, and continuity of care result in inadequate management of both communicable and non-communicable diseases (NCDs) [2]. As the burden of NCDs rises, the push for quality is imperative [3]. The presence of multimorbidity, defined as the occurrence of two or more chronic conditions including those of infectious etiology, compounds this need. In this commentary, we argue that novel methods to evaluate the quality of primary healthcare, such as burden of treatment, must be developed and tailored for use in order for LMICs to achieve equitable outcomes in patients with multimorbidity.

Ideal tools to measure healthcare quality respond to patient expectations in ways that are meaningful to them while also improving their health. The use of patient-reported outcome measures (PROMs) is one way to do this. PROMs reflect patients’ experiences of care and can serve as footprints of patient-centered healthcare reforms. In high-income countries (HICs), their use has been shown to improve quality of care in a patient-centered fashion, and they are even included in some national registries [4, 5].

Despite their successful application in HICs, few PROMS have been effectively used in LMICs, preventing systematic improvement in primary healthcare delivery and perpetuating poor-quality management of chronic conditions. This pattern has led to increased strain on already resource-limited healthcare systems. The presence of both infectious and non-infectious chronic diseases only increases demands by further augmenting the need for long-term, integrated care amongst various specialties and necessitating prevention efforts addressing shared risk factors [6]. Moreover, because LMIC primary healthcare systems evolved to provide short-term, curative programs to treat acute illnesses, they do not contain the infrastructure or organization necessary to provide the multidisciplinary, integrated, and longitudinal care that is required for chronic disease management [3].

Unique epidemiological patterns in the development and distribution of NCDs has created only further challenges for LMIC healthcare systems [7]. This has resulted in NCD onset and mortality at a younger age than in HICs, meaning that healthcare systems must expend more resources over a longer period of time managing the complications of chronic conditions. NCDs also tend to cluster in those with lower socioeconomic status and less education, groups whose capacity to manage extra expenses incurred by chronic diseases is limited [8, 9]. When quality of care is poor, they must deal with the prolonged consequences of their uncontrolled chronic conditions, such as less time spent working or prohibitively high medication costs. This compounds financial losses, driving them, their households, and their communities deeper into poverty and augmenting strain on the healthcare system.

One of the reasons why PROMs have rarely been used as quality indicators in LMICs is that they require rigorous, iterative rounds of evidence gathering in various contexts in order to be valid [10]. To date, this has happened more frequently in HICs [2]. Quality measures in LMICs have instead focused more on healthcare inputs such as medication availability and number of healthcare workers, things are easily measured but not reflective of content of care or patient experience [2]. The result has been a limited amount of meaningful data available to improve quality of care in LMICs [11].

Innovative approaches to develop PROMs that can be used to both measure and track quality improvement in LMICs are essential. Certain aspects of LMIC healthcare systems make successful implementation of PROMs challenging, including paper-based medical record systems and insufficient vital registry systems [5]. Because LMICs are heterogeneous with different clinical environments and medical beliefs, PROMs also need to be flexible and easily adaptable to specific contexts while still maintaining cultural relevance. One potential metric is the burden of treatment, which is defined as how patients’ healthcare workload—things such as medication management and lifestyle changes—affects their health and well-being [12]. Burden of treatment is thought to contribute to suboptimal outcomes in those with chronic conditions. It is especially important in multimorbid patients, as they must do more work to keep their health under control. As a result, adherence to medical treatment is often poor, perpetuating the negative consequences of multiple uncontrolled chronic conditions.

Burden of treatment is unique because of its multidimensional approach. It goes well beyond access to diagnostics and pharmacological treatment. It includes, among other things, the effects that chronic disease management has on personal relationships, the administrative burden it produces, the financial stress it imposes, and its impacts on mental health. These concepts highlight relevant issues at the individual, household and societal level [13]. Because of this, burden of treatment places practitioners and policy makers in the remit of a full understanding of what is required, within and outside of the healthcare system, to manage chronic conditions, including multimorbidity. It also captures how healthcare system barriers that augment patient workload perpetuate high burden of treatment. In LMICs, these barriers are numerous, and the pressures that such barriers exert on engaging with effective care compound with each other towards more obstacles and delayed or poor care. Although these barriers may result in inaccurate diagnoses or insufficient care that initially is less burdensome, this problem only exacerbates the burden of treatment in the long-run. Without timely diagnosis and proper treatment, patients spend more time living in pain or discomfort from their chronic diseases and ultimately receive diagnoses at later stages once complications, sometimes catastrophic, have occurred.

If measured routinely in clinical practice, burden of treatment can potentially be a marker of how healthcare systems are responding to the dual burden of chronic infections and non-infectious diseases as well as the growing burden of multimorbidity. It can serve as a metric that tracks quality improvement and takes into account patient well-being and functionality, shifting efforts from being disease-specific and input-driven to health system-oriented and people-centered [12]. The tools available to measure burden of treatment have been validated mostly in high-income settings and often focus on specific conditions rather than multimorbidity [14]. Although newer tools that are more applicable to LMICs are emerging, their development will require exploratory methods that include qualitative approaches and a focus on multimorbid populations in order to further elucidate which aspects of the burden of treatment are most relevant to LMICs, thus highlighting other aspects of care that may be unique to the LMIC setting, such as access to traditional medicine and the contribution of caregiver burden.

In order to be incorporated routinely into care, tools should be efficient and easily administered by healthcare providers and patients. Because the routine use of PROMs in LMICs is still in its fledgling phases, it will be important to trial various methods of implementation to ensure data collection instruments are accurate and efficient. Innovative solutions, such as the use of electronic spreadsheets or mobile applications, must be tried. Outside of the health sector, the use of time surveys and household-level surveys could be further adapted to inform researchers, policymakers, and practitioners about the burden of treatment beyond patients [15].

Failing to improve quality of primary healthcare in LMICs will result in the development of numerous detrimental downstream effects of poorly managed multimorbidity. Patients will suffer from a poorer quality of life and diminished functional status. Healthcare systems that are already short on resources and money will become even more strained, negatively affecting economies and increasing the equity gap between those living with chronic diseases in LMICs and HICs. The most significant effects will be on those with the fewest resources. Efforts to minimize global inequities in multimorbidity management must be a priority, and they must include the development of outcome measures that are reflective of healthcare quality, meaningful to patients, and relevant to the LMIC setting. Although the use of PROMs is well-established in HICs, developing a tool that is well-suited to be used in primary care settings in LMICs is a novel problem that requires innovative approaches. Because of its multidimensional nature and patient-oriented perspective, measuring burden of treatment is a potential solution. This will help guide policymakers and healthcare workers to systematically implement changes that are important to patients. By improving patient experience and raising their expectations of the healthcare system, quality of care will increase in a meaningful and structured way, minimizing the current inequities in chronic disease outcomes between countries [11]. LMICs will experience less unnecessary mortality, patients will have a better quality of life, economic strain will decrease, and healthcare systems will be better positioned to keep working towards global health equity.
